# Vault changes after cyclopentolate instillation in eyes with posterior chamber phakic intraocular lens

**DOI:** 10.1038/s41598-020-66146-y

**Published:** 2020-06-15

**Authors:** Beatriz Gargallo-Martinez, Jose Javier Garcia-Medina, Elena Rubio-Velazquez, Paulo Fernandes, César Villa-Collar, José M. Gonzalez-Meijome, Ramón Gutierrez-Ortega

**Affiliations:** 1 Department of Ophthalmology, Clínica Novovisión, Murcia, Spain; 2 Departament of Condensed Matter Physics, University of Sevilla, Sevilla, Spain; 3 Departament of Ophthalmology, General University Hospital Morales Meseguer, Murcia, Spain; 4 Department of Ophthalmology and Optometry, University of Murcia, Murcia, Spain; 5 Clinical & Experimental Optometry Research Lab, Center of Physics, University of Minho, Braga, Portugal; 6 Faculty of Biomedicine and Health, European University of Madrid, Madrid, Spain

**Keywords:** Eye diseases, Refractive errors, Vision disorders

## Abstract

Posterior chamber phakic intraocular lens (pIOL) implantation is a common option for correcting moderate-to-high ocular refractive defects. Because this pIOL is implanted on ciliary sulcus, the distance between the back surface of the pIOL and the anterior surface of the crystalline lens, that it is known as vault, should be measured in different conditions to ensure the technique’s safety. Cyclopentolate is a drug that dilates the pupil and relaxes accommodation (cycloplegia). It is often used for different ocular examinations and for other medical purposes. However, there is no evidence of the effect of this drug on vault. This study quantified central vault changes associated with cyclopentolate instillation. We measured the vault under normal conditions (pre-cycloplegic instillation) and after instilling cyclopentolate on 39 eyes of 39 patients with implanted pIOL. Our results suggest that cyclopentolate instillation may induce changes to vault in eyes with implanted pIOL. These changes seem safe and are mainly associated with vault under normal conditions, but also with anterior chamber depth, pupillary diameter and pIOL size.

## Introduction

Visian Implantable Collamer Lens (ICL, STAAR Surgical) is a foldable posterior chamber phakic intraocular lens (pIOL) designed to correct moderate-to-high myopia^1^. The latest models have a central hole that allows better aqueous humour exchange between the anterior and posterior chambers without the need for iridotomy^2^. These models present safe and efficacious results as previous models with no hole^3,4^.

The central vault is defined as the distance between the back surface of the ICL and the anterior surface of the crystalline lens. An appropriate vault has been suggested to be between 250 and 750 µm^5^. An insufficient vault may induce anterior subcapsular cataract due to the contact between the ICL and the anterior lens capsule^1,6,7^. Excessive vaulting can cause angle closure, pupillary blockage or pigmentary dispersion syndrome, which can increase intraocular pressure and, thus, the risk of glaucoma^8–11^. Therefore, it is important to measure this distance to ensure the technique’s safety.

Changes in anterior segment structures appear during accommodation, such as reduced anterior chamber depth (ACD), inducing proximity between the anterior surface of the crystalline lens and the cornea with appearance of synkinetic myosis^12^. The ICL is implanted in the ciliary sulcus. Given its location, dynamic interactions take place between the pIOL and the surrounding structures. Some authors have studied these effects on the vault by accommodative stimulus^13–17^, instillation of pilocarpine for pharmacologically-induced accommodation^13,18^, light-induced pupil constriction^15,17,19^ and mydriasis.^20^ However, no studies have quantified the cycloplegic effect on the central vault.

Cyclopentolate is a drug that dilates the pupil and relaxes accommodation. Preoperative and postoperative evaluations of post-cyclopentolate instillation (by cycloplegic eyedrops) are necessary with patients who have undergone refractive surgery because they allow refraction without accommodation and fundus examination. Cyclopentolate instillation might also be needed after pIOL implantation when other concurrent ocular diseases appear (uveitis, keratitis, retinal detachment and so).

With patients who have undergone pIOL implantation, the cyclopentolate also allows the central and peripherical vault to be measured, but these measurements could be altered secondary to changes in the curvature of crystalline lens surface. We hypothesized that cyclopentolate could have an opposite effect to pilocarpine, but this was not always the case in our clinical practice.

The aim of the current research was to evaluate the effect of cyclopentolate on central vault measurements by studying the dynamic interactions between the ICL and the surrounding structures. We also considered some factors that could influence potential vault changes.

## Results

### Sample characteristics

Thirty-nine eyes of 39 patients who underwent Visian ICL were included. Patients’ demographic and clinical data are summarised in Table 1.

**Table 1:** Patients’ demographic and clinical data.

Eyes/Patients (n)	39/39		
Women (n; %)	29; 74.36%		
Right eye (n; %)	22; 56.41%		
ICL type			
Myopic (n; %)	30; 76.92%		
Myopic toric (n; %)	9; 23.08%		
	**Mean**	**SD**	**Range [minimum, maximum]**
Age (years)	35.67	8.90	[22, 52]
Sphere (D)	−7.83	3.44	[−17.25, −2.00]
Cylinder (D)	−1.07	0.92	[−3.25, 0.00]
Mesopic pupillary diameter (mm)	5.88	1.17	[3, 8]
Intraocular pressure (mmHg)	11.95	2.43	[8, 17]
Pachymetry (µm)	531.90	39.94	[415,617]
ACD (mm)	3.16	0.21	[2.80, 3.64]
W-W (mm)	11.94	0.38	[11.06, 12.60]
ICL power (D)	−9.58	3.40	[−18.00, −3.00]
ICL size (mm)			
12.6 (n; %)	8; 20.51%		
13.2 (n; %)	27; 69.23%		
13.7 (n; %)	4; 10.26%		

SD = standard deviation; D = diopters; ACD = anterior chamber depth; W-W = horizontal diameter white-to-white; ICL = Implantable Collamer Lens; SE = spherical equivalent.

At 6 months post-surgery, the postoperative safety and the efficacy index was 1.12 ± 0.23 and 1.09 ± 0.23, respectively. The spherical equivalent mean improved to −0.03 ± 0.35D in the postoperative examination (P < 0.001). No contact between the posterior pIOL surface and the anterior crystalline lens or angle closure was detected in any eye in this study. Biomicroscopy showed no sign of pigmentary dispersion in any eye. Postoperative intraocular pressure (IOP) was 12.46 ± 2.38 mmHg (ranging from 8 to 18 mmHg) at 6 months post-surgery. There were no significant differences between the mean preoperative and postoperative intraocular pressures (p = 0.218).

### Vault measurements pre- and post-cyclopentolate instillation

**Figure 1:**
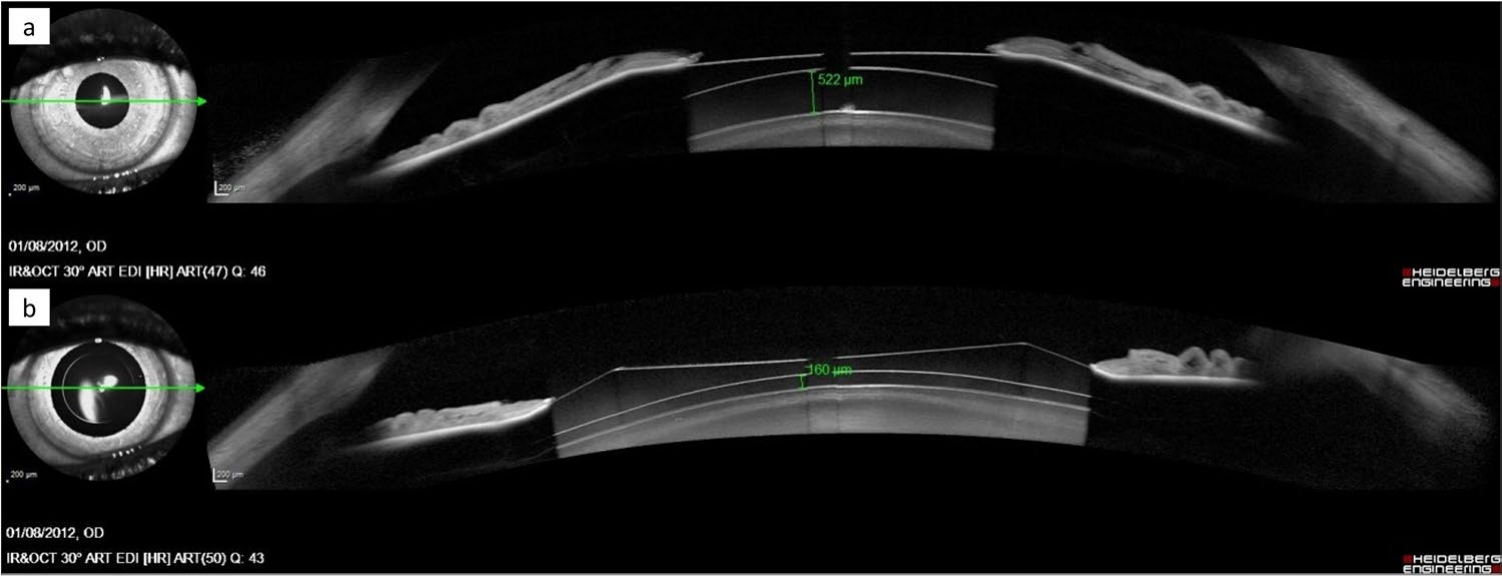
OCT image of the vault measurement. (**a**) normal conditions (top), and (**b**) cycloplegic conditions (bottom). The measurements of the right eye of a 27-year-old patient with a 5-mm pupil, 3.4-mm ACD, 11.80-mm W-W, 13.20-mm ICL and -10.50-diopter ICL power shows a decreased vault that resulted from cycloplegic drug instillation.

**Figure 2:**
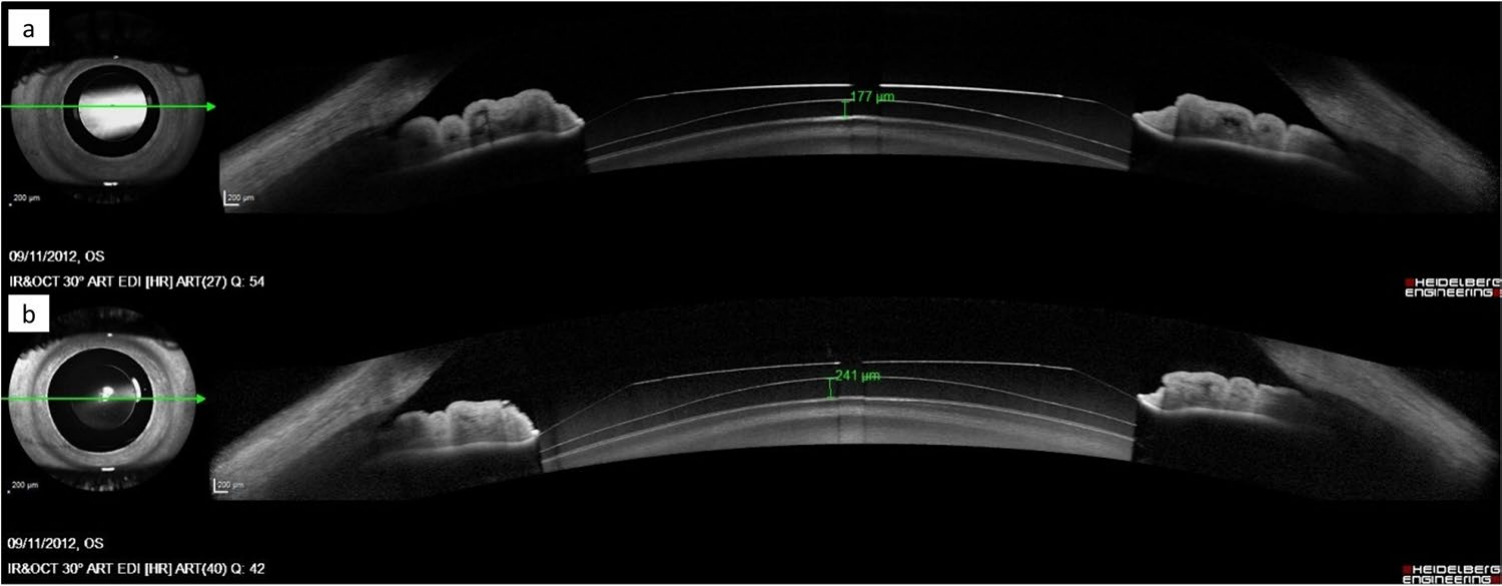
OCT image of vault measurement (**a**) under normal conditions (top), and (**b**) cycloplegic conditions (bottom). The measurements of the left eye of a 29-year-old patient with a 7.5-mm pupil, 2.98-mm ACD, 11.70-mm W-W, 12.60-mm ICL and -12.50-diopter ICL power shows an increase in vault that resulted from cycloplegic drug instillation.

The mean vault under normal conditions (Nvault, mean: 534.6 ± 339.6 µm, ranging from 50 to 1407 µm) was significantly (p < 0.001) higher than the mean vault under cycloplegic conditions (Cvault, mean: 451.6 ± 259.6 µm, ranging from 63 to 1096 µm) (Fig. 1). However, the opposite change was observed in some patients (Fig. 2). Cvault dropped more than 20 µm the Nvault in 23 eyes (59%), increased more than 20 µm in eight eyes (20.5%) and was similar (change between −20 µm and +20 µm) in eight eyes (20.5%).

### Relationship between vault change (∆vault = Cvault-Nvault) and Nvault

**Figure 3:**
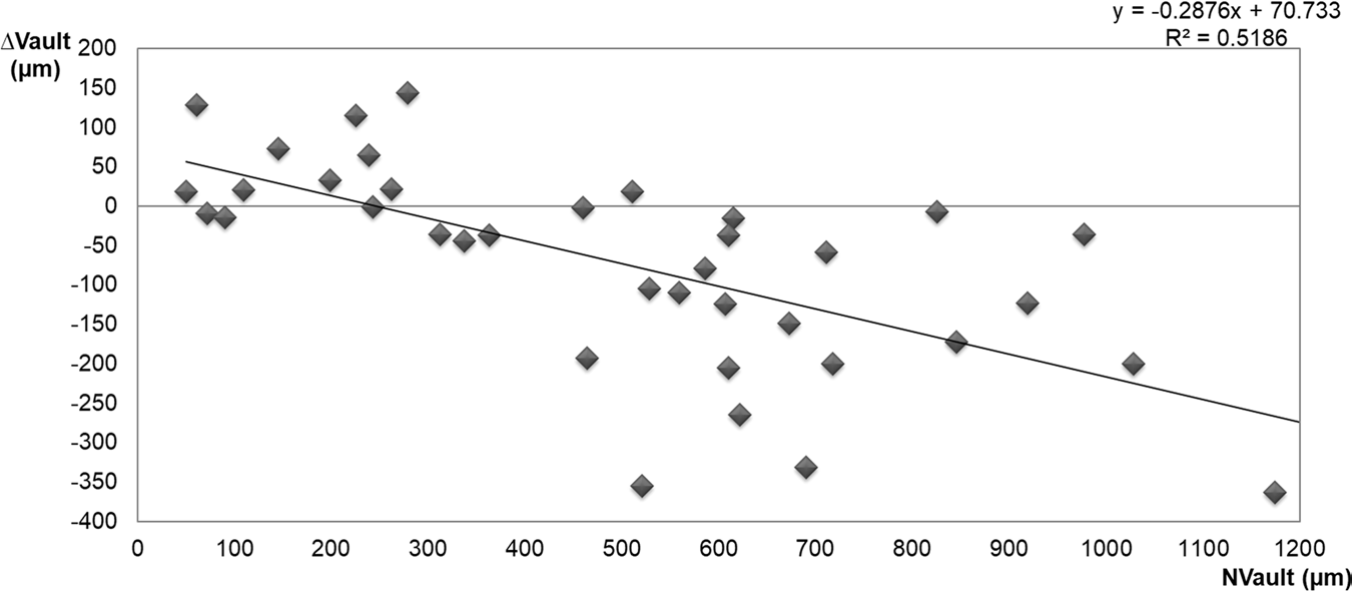
Scatterplot representing the linear correlation between the normal vault (Nvault) and ∆vault resulting from cycloplegic drug instillation. ∆vault = Cvault-Nvault. Nvault = vault under normal conditions. Cvault = vault after cyclopentolate instillation.

∆vault correlated significantly with Nvault (Pearson’s coefficient correlated r = −0.720, p < 0.001). (Fig. 3). In the eyes with Nvault <511 µm, ∆vault was significantly lower (*P* < 0.001) than in the eyes with Nvault ≥511 µm (+16.9 ± 76.7 µm vs. −168.8 ± 115.1 µm, respectively).

Nvault also correlated significantly with CVault (r = 0.932, p < 0.001) and ACD (r = 0.399, p = 0.010).

### Relationship between vault change (∆vault = Cvault-Nvault) and ACD

**Figure 4:**
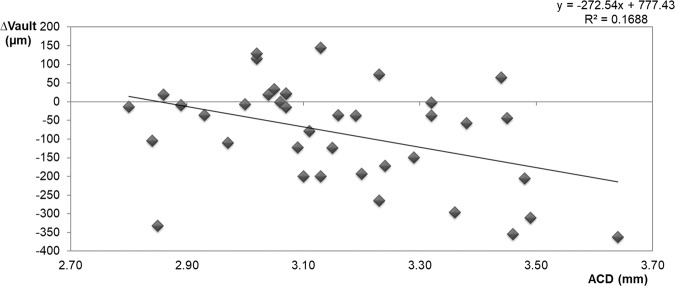
Scatterplot representing the linear correlation between the ACD and ∆vault resulting from cycloplegic drug instillation. ∆vault = Cvault-Nvault. Nvault = vault under normal conditions. Cvault = vault after cyclopentolate instillation.

∆vault correlated significantly with ACD (Pearson’s correlation coefficient, r = −0.408, p = 0.010) (Fig. 4). In the eyes with ACD < 3.13 mm, ∆vault was significantly (p = 0.014) lower than in the eyes with ACD ≥ 3.13 mm (−34.7 ± 115.0 µm vs. −139.9 ± 138.9 µm).

### Relationship between vault change (∆vault = Cvault-Nvault) and Pupillary diameter

**Figure 5:**
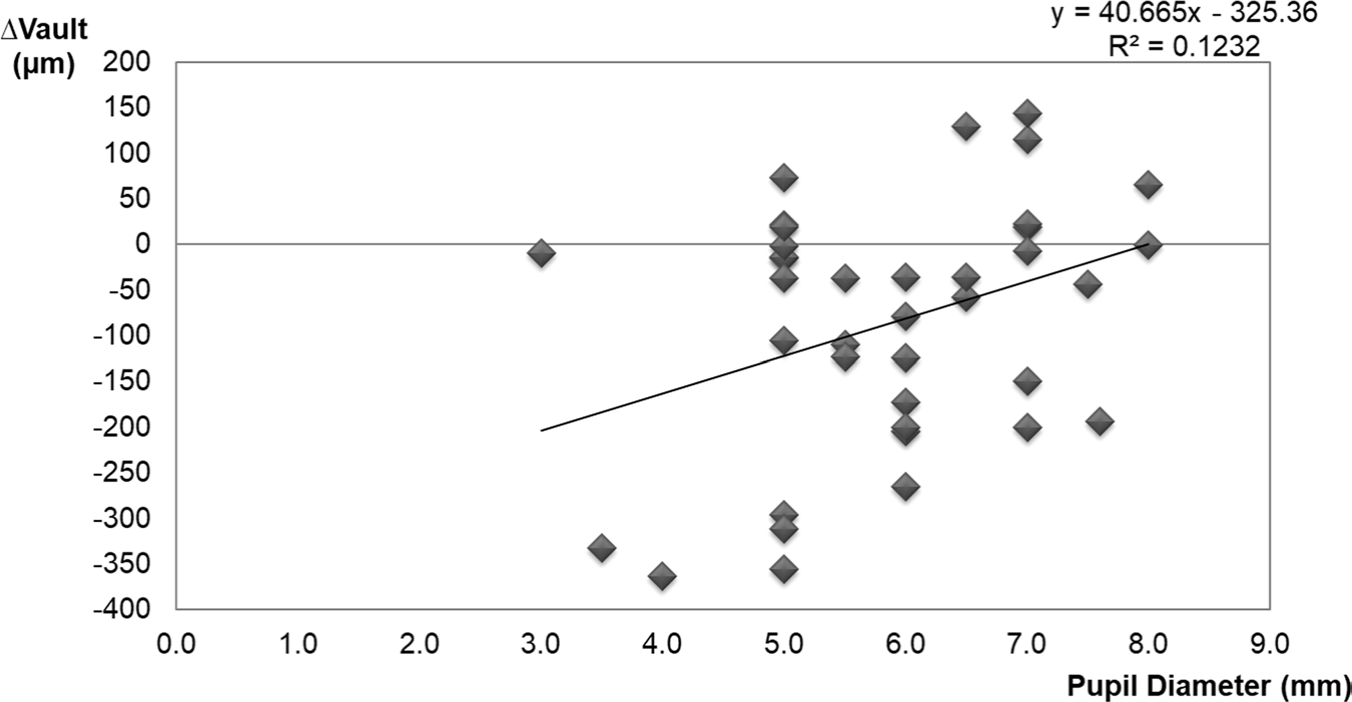
Scatterplot representing the linear correlation between the ∆vault and pupillary diameter resulting from cycloplegic drug instillation. ∆vault = Cvault-Nvault. Nvault = vault under normal conditions. Cvault = vault after cyclopentolate instillation.

∆vault also significantly correlated with the pupillary diameter (Pearson correlation coefficient, r = 0.351, p = 0.039) (Fig. 5). ∆vault in the eyes with a pupil <6.00 mm was significantly (p = 0.007) more negative than in eyes with a pupil ≥6.00 mm (−128.2 ± 133.6 µm vs. −10.7 ± 107.3 µm, respectively).

### Relationship between vault change (∆vault = Cvault-Nvault) and ICL characteristics

**Figure 6:**
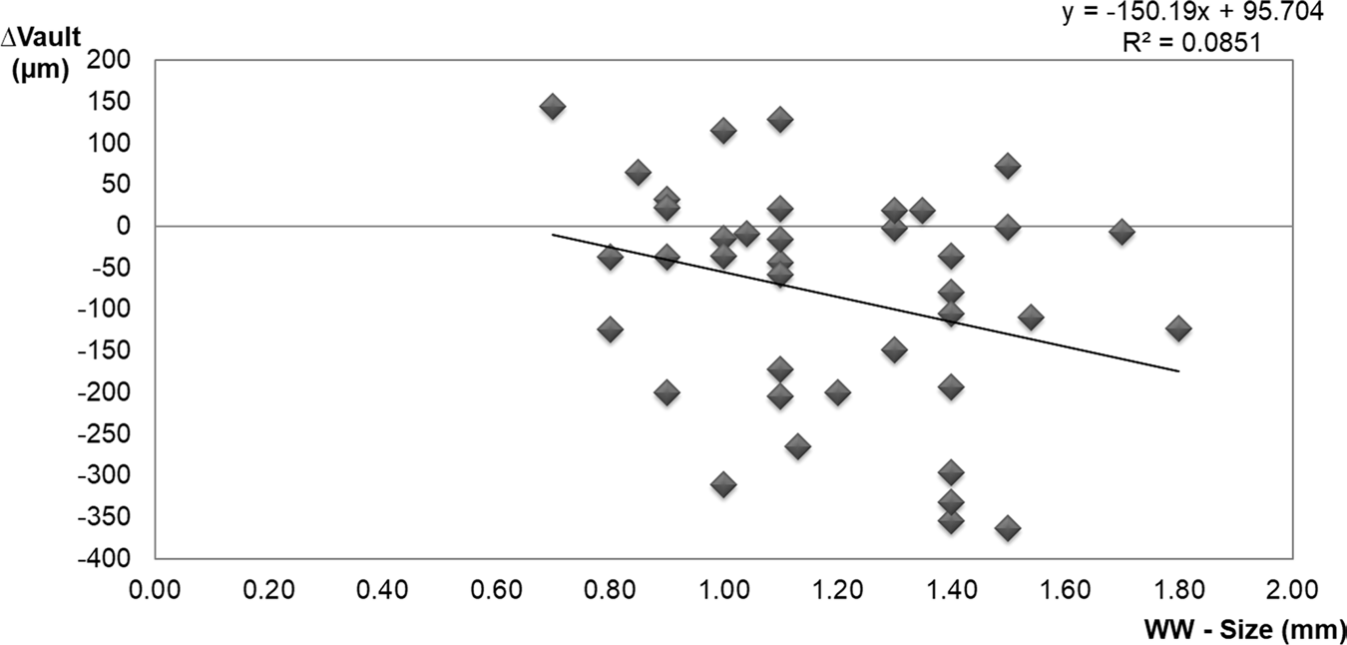
Scatterplot representing the linear correlation between ∆vault and the difference in WW-ICL size resulting from cycloplegic drug instillation. ∆vault = Cvault-Nvault. Nvault = vault under normal conditions. Cvault = vault after cyclopentolate instillation. WW = White-to-white.

∆vault did not significantly correlate with WW-size i.e. the difference between horizontal white-to-white diameter and ICL size (Pearson’s correlation coefficient, r = −0.292, p = 0.071) (Fig. 6). However, in the eyes with WW-size <1.1 mm, ∆vault was significantly (p = 0.026 by the t-test) lower than in the eyes with WW-size ≥1.1 mm (−36.7 ± 117.8 vs. −131.8 ± 138.8 µm, respectively).

**Figure 7:**
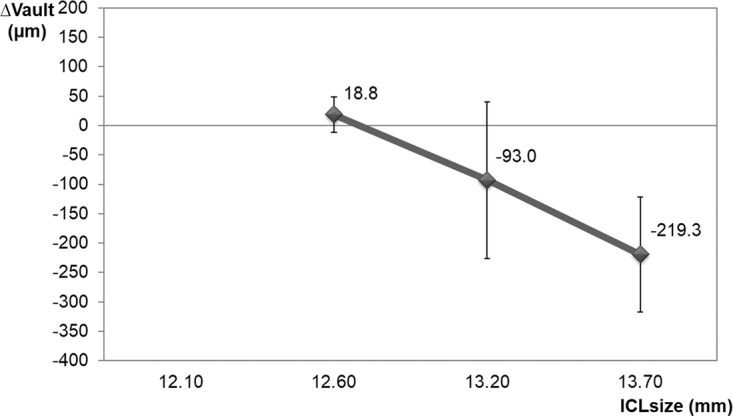
Mean ∆vault values resulting from cyclopentolate instillation for each ICL size. ∆vault = Cvault-Nvault. Nvault = vault under normal conditions. Cvault = vault after cyclopentolate instillation. ICL = implanted collamer lens.

Different ICL sized groups showed a distinct ∆vault (p = 0.009) (Fig. 7). The smallest ICL size (12.60 mm) had a mean ∆vault of +18.8 ± 85.7 µm, which was significantly lower and more positive than the largest ICL size (13.70 mm) with a mean ∆vault of −219.3 ± 97.5 µm (p = 0.012). The ICL size 13.2 group presented no significant differences in ∆vault to other ICL size groups (p = 0.090 and p = 0.171, respectively).

Nvault also differed in each ICL size (p = 0.035). The smallest ICL size presented the lowest Nvault (296.3 ± 262.2 µm), followed by 13.2 size (567.6 ± 333.2 µm), while the largest ICL size showed the highest Nvault (789.0 ± 300.3 µm).

pIOL power did not significantly correlate with ∆vault (p > 0.05).

### Relationship between vault change (∆vault = Cvault-Nvault) and all parameter measured

Age and pachymetry did not significantly correlate with ∆vault (p > 0.05). The multiple stepwise linear regression results showed that the most relevant variable to ∆vault was Nvault (p < 0.001, partial regression coefficient B = −0.271; standardised coefficient Beta = −0.677), followed by pupillary diameter (p = 0.032, partial regression coefficient B = 29.05, standardised coefficient Beta = 0.251). The remaining parameters showed no statistical significance into multivariate analysis so they were removed. The final model obtained was good and predicted more than 50% of vault change (R = 0.756, R^2^ = 0.571, adjusted R^2^ = 0.547, p < 0.001). Accordingly, vault decreased after cycloplegic instillation as Nvault increased and pupil diameter belittled.

## Discussion

Vault measurement after ICL implantation is determined to ensure surgical safety. As several parameters affected the vault after surgery^21–24^ and this can change with time^25–28^, accurate objective measurements are required under different conditions in the short and long terms to understand the dynamic interactions between the ICL and the surrounding structures.

The current results suggest that cycloplegic eyedrops have different effects on vault measurements, and several parameters may influence such changes. Figures 1 and 2 illustrate two cases with different results.

Previous studies have reported a relation among ocular size, vault degree and ICL size. While eyes with a deep anterior chamber and/or a high W-W have higher vaults with a bigger lens size (this case tends to oversize the ICL), eyes with a shallow anterior chamber and/or low W-W have lower vaults with a smaller lens size (undersizes the ICL)^29,30^. Similarly, the current results suggest that ∆vault depends on some ocular parameters. The eyes with a high Nvault, a deep anterior chamber or a bigger lens size showed a similar and a more marked reduction in vault with the cyclopentolate effect (cycloplegic eyedrops reduced Nvault). In contrast, the eyes with low Nvault, a narrow anterior chamber or a smaller lens size presented a less marked reduction or a higher vault value after instilling the cycloplegic drug.

The multiple regression analysis showed that the most important parameter for vault change after instilling cycloplegic eyedrops was vault under normal conditions (Nvault). Therefore, the cycloplegic effect on vault could be explained by changes in ciliary muscle and horizontal lens compression. Lewis *et al*.^31^ showed that maximal ciliary muscle thickness increased with the accommodative response and decreased under cycloplegic conditions. As the ICL footplate is supported in the sulcus, changes in ciliary muscle tone may modify vault. Zhu *et al*.^20^ noted that the distance between the corneal endothelium and the ICL significantly increased after pharmacological mydriasis (the ICL moved backwards). They hypothesised that this phenomenon was due to enlargement of the sulcus-to-sulcus distance after pharmacological ciliary muscle relaxation. Therefore, if the cycloplegic eyedrops reduce horizontal compression, vault should reduce. This would explain why the vault with oversized ICLs was affected more by a cycloplegic drug than undersized ICLs. An oversized ICL produces greater lens compression in the ciliary sulcus which leads to a high degree of vaulting^22^, which is affected more by the cycloplegic drug (the cycloplegic vault decreased further, as in big eyes with a deep ACD). Our results agree with the case reported by Bechmann *et al*.^32^ These authors showed a patient with large eyes (ACD > 3.7 mm) with increased vaulting during the accommodative stimulus and decreased vaulting when instilling a cycloplegic drug. However, an undersized ICL led to a situation in which the ICL footplate was not well wedged in the ciliary sulcus. This resulted in lack of horizontal compression^22^ and low vaulting, thus cycloplegic agent would affect the vault less (Δvault came close to 0, as in eyes with a narrow ACD).

As pupil diameter was the second most important variable for vault change according to the multiple linear regression analysis, pupil variations and their relationship with vault were also analysed in our study. Some authors have reported that under photopic conditions with pupillary constriction, the distance between the ICL and the crystalline lens significantly decreased^13,15,17,19^. This has been explained by the vertical compression produced by the iris^13,17^. If the vault is lower when the pupil is constricted (with the photomotor reflex), it is understandable that the cycloplegic drug could cause the opposite effect, and vault would be similar or higher with a dilated pupil. However, the present results indicated the pupillary diameter effect on ∆vault under mesopic conditions, we showed that small pupils had the opposite effect to what was expected (the vault decreases with cycloplegia), and large pupils were less affected by cycloplegic drops (the cycloplegic vault is similar or higher). Numerous interactions occur between the ICL and the eye, and the ICL and the vault may be influenced differently by the iris musculature, depending on the lighting conditions. We believe that the pupillary sphincter induces centripetal force on the ICL, which increases horizontal compression to cause higher vaults under mesopic conditions and, therefore, the cycloplegic drug eliminates this pressure and reduces the vault. This effect would be independent of the vertical compression produced by photomotor reflex.

Accommodation causes changes to the anterior segment, i.e., a decreased ACD due to the proximity of the anterior surface of the crystalline lens to the cornea by the forward bulging of the anterior lens surface and a smaller pupillary diameter^12,33,34^. If the anterior surface of the crystalline lens approaches the cornea during accommodation, we expect the central vault to decrease^13–15,17,20^ and, therefore, the distance between the ICL and the crystalline lens should increase when instilling a cycloplegic drug, and accommodation is no longer active. This would explain our results in those eyes in which a small ICL (with no horizontal compression) and/or a low degree of vaulting had a similar or slightly higher vault when instilling a cycloplegic drug. Maldonado *et al*.^35^ reported a case with similar findings to our study. A patient implanted with a phakic IOL (ICL V3 model; 11.5 mm) had a normal vault postoperatively in which the ICL came completely into contact with the anterior pole of the crystalline lens when 2% pilocarpine (pharmacologic accommodation) was instilled; 24 hours after instilling 1% cyclopentolate, the ICL vault increased to 132 μm. Zhu *et al*.^20^ showed a slight vault increase of 46 µm after pharmacologic mydriasis with Mydrin-P, which contains 0.5% (w/v) tropicamide and 0.5% (w/v) neophryn. These authors found this increase 6 months after Visian V4c implantation with 462.5 ± 162.7 µm of vault prior to dilation, which is comparable to our results (our patients with Nvault < 511 µm presented a slightly increased vault after cycloplegic eyedrops). Despite an increase in vault being expected as a result of accommodation paralysis, the opposite effect shown (some lenses reducing the vault after cycloplegic instillation) cannot be explained by a change in crystalline lens. Zhu *et al*.^20^ also found that the vault decreased in 11 eyes of 8 patients (6.4%).

There are some methodological differences between the research performed by Zhu *et al*. and ours that could justify the different results, at least in part. Firstly, the eyedrops used in both studies were not the same (combination of tropicamide and neophryn *versus* cyclopentolate), which may have cause different ocular effects. Secondly, they mostly considered both eyes of one same patient for the analysis instead of one eye per patient, as in the present study. Thirdly, they considered only one measurement per vault estimation, whereas we considered the mean of three measurements. Fourthly, the illumination conditions for vault measurements are not described in their study, which have been demonstrated to affect both the ICL position and the central vault^17,19^. The basal vault measure (Nvault) in our study was performed under mesopic conditions. Fifthly, they did not study the association between ∆vault and other possible influencing factors as we did. Finally, the authors used Pentacam, whereas we employed anterior segment OCT to measure vault values. As recently shown by Wang *et al*.^36^, Pentacam and anterior segment OCT cannot be interchanged to estimate pIOL vault.

Our results also suggest that using cyclopentolate eyedrops is safe in all cases. In the eyes with high Nvaults, cyclopentolate was associated with a decreased vault (Fig. 3) and did not increase the risk of pigmentary glaucoma if we consider the following facts: (a) no signs of pigmentary dispersion were detected in any eye during the 6-month follow-up; (b) postoperative IOP at the 6-month follow-up remained unchanged and did not go beyond 21 mmHg in any eye. In the eyes with low Nvault, no contact between the posterior pIOL surface and the anterior crystalline lens was detected in any eyes before or after cyclopentolate instillation. Cyclopentolate was associated with increased vault in these cases (Fig. 3), which minimises the risk of cataracts developing after ICL implantation. Thus, we can speculate that these eyedrops could be used as treatment in the eyes where vault is 0 (contact between pIOL and the crystalline lens) while waiting for ICL exchange to be performed.

All these considerations allow us to conclude that cyclopentolate instillation is not only safe 6 months after ICL implantation, but also offers a protective effect against pigmentary glaucoma (because this drug seems to decrease ∆vault in the eyes with high Nvault) and cataract formation (because it seems to increase ∆vault in the eyes with low Nvault).

Additional studies are necessary to confirm the current findings in order to better understand the reaction of the ICL under all kinds of conditions. As accommodation and the cycloplegic drug change ICL vault, it is reasonable to measure this distance under the effect of pilocarpine and cyclopentolate during the postoperative follow-up to ensure surgical technique safety. It is also important to define the different conditions needed to take objective vault measurements as these conditions may affect them. As supported by our results, cyclopentolate may induce artifactual results on vault estimations, which should be taken into account when examining eyes with pIOL implantation.

This is the first study to analyse the effect of cycloplegic eyedrops on ICL vault. Although the sample is relatively small (n = 39), the selection of only one eye per patient and considering the mean of three measurements per vault estimation confer our results reliability. Another issue is that we did not measure axial length preoperatively because it was not necessary for pIOL diameter or power calculation as described in methods section. However, we consider that analysing the effect of axial length on ICL behaviour could be interesting for further studies.

In conclusion, the present results suggest that cyclopentolate eyedrops may change the mean central vault in eyes with pIOL implantation. This effect is related to Nvault, ACD, pupillary size and ICL size. Our results also indicate that cyclopentolate instillation is safe in these eyes.

## Methods

### Study characteristics

This prospective study included consecutive patients who underwent Visian ICL implantation at Clinica Oftalmologica Novovision in Murcia, Spain. The study protocol was conducted according to the Declaration of Helsinki and was approved by the Ethics Committee from Clinica Oftalmologica Novovision, Murcia, Spain. Informed consent was obtained from all the patients.

### Preoperative examination

A complete ocular examination was performed preoperatively to determine indications for ICL implantation, including topography, pachymetry, ACD measured using the Oculus Pentacam (Germany), horizontal white-to-white diameter (W-W) measured by Allegro Topolyser (WaveLight), pupillometry under mesopic conditions (Colvard Pupillometer), manifest and cycloplegic refractions, best-corrected visual acuity (BCVA), central corneal endothelial cell count (ECC) (Specular Microscope CEM-530, Nidek), slit-lamp biomicroscopy, intraocular pressure (IOP), and dilated fundus examination. The parameters of the ICL (power and size) were calculated using the software v4.08 of Staar Surgical S.L. (available online at https://ocos.staarag.ch/). The keratometry, the ACD and the pachimetry were used to estimate the power of the pIOL. The W-W and the ACD were used to calculate the size of the pIOL. Axial length was not necessary for the calculation of pIOL power or size.

### Visian ICL implanted

The implanted pIOLs were Visian ICL model V4C (with a central hole) and emmetropia was targeted. ICL size was determined for each patient according to the manufacturer’s recommendations based on W-W and ACD. All surgical actions were performed by the same surgeon (R.G.O.) without complications, who implanted and horizontally oriented all the lenses at the Novovision Ophthalmologic Clinic, Murcia, Spain.

### Inclusion and exclusion criteria

The inclusion criteria in this study were age >18 years, stable myopia, ACD ≥ 2.8 mm, ECC > 2,000 cells/mm^2^ and postoperative follow-up after 6 months of surgery. Only one eye per patient was selected. If both eyes of the same patient were eligible, a randomly selected eye was chosen. Patients with a history of any disease that might affect anterior segment structure function were excluded.

### Postoperative examination and vault measurements

Six months after ICL implantation, the same series of examinations was repeated. Assessments of surgery outcomes were performed by the safety index (postoperative-BCVA/preoperative-BCVA) and the efficacy index (uncorrected-visual-acuity-postoperative/BCVA-preoperative). Two different central vault measurements were also estimated by anterior segment optical coherence tomography (OCT) with the device Spectralis SD-OCT (Heidelberg Engineering, Heidelberg, Germany) under mesopic conditions. Firstly, vault estimations were measured under normal conditions (Nvault), with the patient focusing on an internal fixation point. Then instillations of one drop of cyclopentolate at 10-minute intervals were applied (3 instillations in all). Forty minutes after the first instillation, a second vault estimation was performed with cycloplegia (Cvault). Vault measurements were taken perpendicularly to the ICL and centred on the hole. The same technician (B.G.M.) assessed three different vault measurements under the two distinct conditions (pre and postcycloplegia) and calculated the mean vault value in each case. The difference between the mean Cvault and the mean Nvault (Cvault-Nvault) was calculated for each eye and referred to as ∆vault.

### Statistical analysis

A statistical analysis was performed using the SPSS software, version 19.0 (SPSS Inc.). Data distribution normality was confirmed by the Shapiro-Wilk test. The difference between the Nvault and Cvault measurements was studied by a paired-samples Student’s t-test. ∆vault was correlated with Nvault, ACD, pachymetry, pupillary diameter, ICL size and power, difference between W-W and ICL size (WW-size) and patient’s age using the Pearson coefficient correlation test. In order to evaluate the effect of the different variables on ∆vault, the sample was divided into two groups based on the dependent variables. The cutoff values (Nvault, 511 µm; ACD, 3.13 mm; pupil, 6.00 mm; W-W size, 1.1 mm) were arbitrarily set based on the 50^th^ percentile value. The mean difference of these mentioned variables between these created groups was also studied by the independent-sample Student’s t-test. The mean difference of ∆vault between ICL sizes was analysed by an ANOVA test, followed by the Scheffe *post hoc* test. Multiple linear regression was finally performed. *P* ≤ 0.05 was considered statistically significant.
